# Influence of the force magnitude of fixed functional appliances for class II subdivision 1 treatment—a cephalometric study

**DOI:** 10.1007/s00056-023-00455-5

**Published:** 2023-03-06

**Authors:** Hisham Sabbagh, Aladin Sabbagh, Mila Janjic Rankovic, Christine Huber, Andrea Wichelhaus, Lea Hoffmann

**Affiliations:** 1 Department of Orthodontics and Dentofacial Orthopedics, University Hospital, LMU Munich, Goethestr. 70, 80336 Munich, Germany; 2 Private Orthodontic Practice, Erlangen, Germany; 3 Private Orthodontic Practice, Munich, Germany

**Keywords:** Class II malocclusion, Fixed orthodontic appliances, Class II therapy, Orthodontic treatment, Force-generating springs, Klasse-II.1-Malokklusion, Festsitzende kieferorthopädische Apparaturen, Klasse-II.1-Behandlung, Kieferorthopädische Behandlung, Druckerzeugende Federn

## Abstract

**Purpose:**

To investigate the skeletal and dental effects of a hybrid fixed functional appliance (FFA) used with different force magnitudes for class II subdivision 1 treatment.

**Methods:**

Treatment records from 70 patients were evaluated: 35 patients were treated with a FFA with standard activation (SUS group) and 35 with a FFA with an additional force-generating spring (TSUS group). Two control groups were matched from the American Association of Orthodontists Foundation (AAOF) Craniofacial Growth Legacy Collection for comparison with the two treatment groups to determine skeletal and dental treatment effects. The cephalometric parameters at T0 (before treatment) and T1 (before debonding) were assessed using the Munich standard cephalometric analysis and by the sagittal occlusal analysis (SO) according to Pancherz. Data were analyzed statistically using SPSS.

**Results:**

No statistically significant difference for any cephalometric parameter was observed between the SUS and TSUS groups concerning the measurements at T0 and T1. Both treatment groups exhibited an effective class II therapy mainly due to a significant reduction in SNA, and ANB and an increase in SNB. In contrast to the control group, as the result of treatment a skeletal class I was achieved.

**Conclusion:**

No significant statistical differences were observed between the patient group treated with the FFA with standard activation (SUS) and those treated with an additional spring (TSUS) regarding the cephalometric parameters investigated. Both variants were equally effective in treating class II division 1 malocclusions.

## Abstract (de)

**Zielsetzung:**

Ziel dieser Studie war die Untersuchung skelettaler und dentaler Effekte festsitzender Klasse-II-Mechaniken (FM), die mit unterschiedlichen Kraftgrößen bei der Behandlung von Klasse-II.1-Dysgnathien eingesetzt wurden.

**Methoden:**

Die Behandlungsunterlagen von 70 Patienten wurden ausgewertet. 35 Patienten wurden mit einer FM mit Standardaktivierung (SUS-Gruppe) und 35 mit einer FM mit zusätzlicher kraftgenerierender Feder (TSUS-Gruppe) behandelt. Zwei Kontrollgruppen wurden aus der AAOF (American Association of Orthodontists Foundation) Craniofacial Growth Legacy Collection zum Vergleich mit den beiden Behandlungsgruppen zusammengestellt, um die therapiebedingten skelettalen und dentalen Effekte zu bestimmen. Die kephalometrischen Parameter bei T0 (vor der Behandlung) und T1 (vor dem Debonding) wurden mit der Münchner Standard-Analyse und der sagittalen Okklusionsanalyse (SO) nach Pancherz analysiert. Die Daten wurden mit SPSS statistisch ausgewertet.

**Ergebnisse:**

Zwischen der SUS- und der TSUS-Gruppe wurde kein statistisch signifikanter Unterschied für die untersuchten kephalometrischen Parameter hinsichtlich der Messungen bei T0 und T1 festgestellt. Beide Behandlungsgruppen zeigten eine Korrektur der skelettalen Klasse II, die hauptsächlich auf eine signifikante Verringerung der SNA und ANB und eine Erhöhung der SNB zurückzuführen war. Im Gegensatz zur Kontrollgruppe wurde als Ergebnis der Behandlung eine skelettale Klasse I erreicht.

**Schlussfolgerung:**

Hinsichtlich der untersuchten kephalometrischen Parameter wurden keine statistisch signifikanten Unterschiede zwischen der festsitzenden Klasse-II-Mechanik mit Standardaktivierung (SUS) und der festsitzenden Klasse-II-Mechanik mit einer zusätzlichen kraftgenerierenden Feder (TSUS) festgestellt. Beide Varianten waren bei der Behandlung von Klasse-II.1-Dysgnathien gleich wirksam.

## Introduction

Class II division 1 malocclusion is defined by a distal molar relationship and an increased overjet [31] and belongs to the most frequent malocclusions with a worldwide prevalence between 15 and 25% [1, 32]. Besides esthetic and functional reasons [36–38], the increased risk of traumatic dental injury represents a particular indication for early treatment of class II division 1 malocclusions in children [5, 6, 17, 30].

Available treatment approaches are diverse and can be classified according to the type of appliance (removable or fixed), treatment timing (one-step or two-step), or whether extractions or surgical interventions are planned [37]. The treatment approach depends on the severity of the malocclusion, the patient’s age and residual growth potential, compliance, and the experience and individual preference of the clinician.

Fixed functional appliances belong to the most frequently used appliances for nonsurgical treatment of class II malocclusions [22]. More than 50 different appliances are commercially available [24]. Based on the force system used, fixed functional appliances (FFAs) can be classified into rigid, flexible, and hybrid [33]. Flexible FFAs like the Jasper Jumper [16] are characterized by an intermaxillary spring that allows almost unrestricted mandibular movements. On the other hand, rigid FFAs like the Herbst appliance [28] work on the basis of a telescopic mechanism that forces the mandible into a forward position and, therefore, only allows restricted mandibular movements. Hybrid appliances, like the Sabbagh universal spring [20], combine the telescopic mechanism with an intermaxillary spring.

The treatment effects of FFAs are well studied [41]. Generally, class II correction is achieved by skeletal and dental effects. The skeletal effects result from restricted maxillary growth and remodeling of the mandibular condyle and glenoid fossa [12, 15, 23, 26, 41], whereas the dental effects are caused by maxillary molar distalization, mandibular molar mesialization, and protrusion of lower incisors [13].

However, previous literature is not consistent on the extent and distribution of dental and skeletal effects [23, 41] and the significance of long-term effects of growth modification in comparison to natural growth is still controversial [12, 15]. In particular, the influence of the force magnitude on the dental and skeletal effects has not been clarified to date. Available systematic reviews on the topic concluded that further studies are required to investigate the influence of particular features of the used FFAs including the appliance design and additional elements, such as force-generating springs [21, 41].

Therefore, the aim of the present study was to investigate the differences in treatment effects of a hybrid FFA with and without an additional force-generating spring, based on dental and skeletal cephalometric measurements. The null hypothesis was that FFAs with additional force-generating springs show more dental and skeletal effects than FFAs without.

## Patients and methods

Sample size was calculated based on published data for class II treatment with FFAs [8, 9, 18, 39] using the software G*Power (version 3.1.9.6 for macOS [14]). Effect size was calculated for the parameter ANB (mean H0 = 0, mean H1 = −2.78, SD = 1.61 [39]; Cohen’s d = 1.73) and a two-tailed t‑test was used assuming α = 0.05 for a power of 0.95, resulting in a total sample size of 7. To account for a potentially smaller effect size, a plot was calculated with a small effect size of 0.6, yielding a sample size of 20 patients per group for a power of 0.95. The study protocol was approved by the ethics committee of the Ludwig Maximilian University of Munich (ref. No. 21-0113).

Dental and skeletal treatment effects of two different groups treated with a hybrid FFA (SUS^2^, Sabbagh Universal Spring 2, Dentaurum, Mannheim, Germany) were investigated: (1) patients treated with the standard activation of 2.5 N/side (SUS group); (2) patients treated with an additional spring of 3.0 N/side (TSUS group).

Diagnostic records of the two treatment groups (SUS/TSUS) were retrospectively collected between 2017 and 2020 from a private orthodontic practice (Erlangen, Germany). Data included, age, gender, treatment duration, appliance used, and cephalometric images at two different time points, T0 (before treatment) and T1 (before debonding). Patients between 11 and 15 years of age who had passed the pubertal growth peak (cervical vertebral maturation stage [CVMS] analysis [4], stage III), and who presented with a skeletal class II (according to ANB and Indiv. ANB [27]) were treated with a hybrid FFA and were included in the study. Table 1 summarizes the inclusion and exclusion criteria. Out of 110 patients, 35 patients in each group (SUS/TSUS) were included according to these criteria.

**Table 1:** Inclusion and exclusion criteria Ein- und Ausschlusskriterien

Inclusion criteria	Exclusion criteria
Skeletal class II (ANB > individual ANB + 1°)	Previous orthodontic treatment
Patients between 11 and 15 years of age	Tooth agenesis
Cervical vertebral maturation stage (CVMS) III	Tooth extractions
Completed treatment with class II fixed functional appliance (SUS/TSUS)	Craniofacial anomalies
Complete diagnostic records	Vertical growth pattern

*SUS* standard activation, *TSUS* activation with an additional force-generating spring

To compare the dental and skeletal effects of the FFAs, two nontreated control groups (one for the SUS group and one for the TSUS group) were obtained from the American Association of Orthodontists Foundation (AAOF) Craniofacial Growth Legacy Collection after matching according to following parameters: (1) matched age at the time of the first cephalometric record (T1) in the respective treatment group (±6 months), (2) same sex, (3) skeletal class II (ANB > individual ANB + 1°), (4) no previous orthodontic treatment, (5) patients without tooth extraction and/or tooth agenesis.

### Treatment protocol

**Fig. 1:**
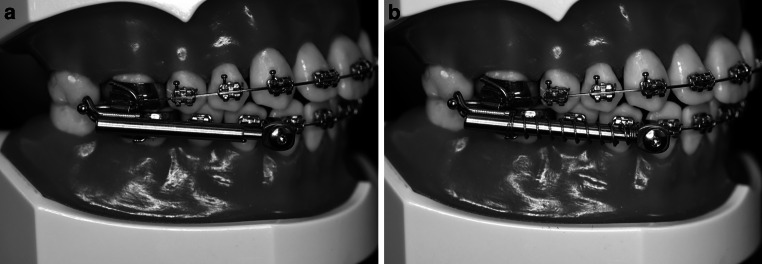
Hybrid fixed functional appliance (FFA) without (SUS, **a**) and with (TSUS, **b**) an additional spring Hybride FFA („fixed functional appliance”) ohne (SUS, **a**) und mit (TSUS, **b**) Zusatzfeder

All patients received an orthodontic straight-wire appliance (0.018″ slot, MBT prescription) with orthodontic bands on the upper first molars. First, teeth were leveled and aligned until a 0.016″ × 0.022″ stainless steel archwire could be inserted and no crowding or spacing was left. Subsequently, the hybrid FFA was inserted. Doing so, the appliance was attached to the orthodontic bands on the upper first molars and on the lower archwire between the canines and the first premolars on both sides (Fig. 1). Figure-eight ligatures were used in the upper and lower arch to avoid spacing. In addition, cinch-back bends were applied in the lower jaw and a torque of 10° was applied for the upper incisors to control their inclination. Initial activation force was 2.5 N in the SUS group and 5.5 N in the TSUS group, which was controlled by ensuring that the inner telescope did not protrude from the outer telescope in closed mouth position (full activation of the inner force spring). Otherwise a spacer ring was applied to achieve full activation. During the control appointments (every 6 weeks), activation with spacer rings (1.0 mm) was performed to restore full activation until edge-to-edge position of the upper and lower incisors was achieved.

### Cephalometric evaluation

Lateral cephalograms were taken before treatment (T0) and before debonding (T1). The radiographs were individually calibrated and traced by one operator (C.H.). Two cephalometric analyses were performed for each patient: the standard analysis of the orthodontic department of the university hospital and the sagittal occlusion analysis according to Pancherz [29]. Standard analysis was performed digitally using the software FR-Win (Computer Konkret Dental Software, Falkenstein, Germany). For the Pancherz analysis, the digitally calibrated images were printed and manually traced, where the OL/OLp reference grid was transferred from the first to the second radiograph. The operator had been trained and calibrated on an independent sample of lateral cephalograms. Furthermore, for the evaluation of the reliability of the cephalometric measurements performed by the operator, 10 lateral cephalograms were randomly chosen and retraced after a wash-out period of 4 weeks. An averaged contour line was used for structures that showed a double contour in the radiograph. The cephalometric variables used and their definitions are shown in Table 2.

**Table 2:** Definitions of the cephalometric variables investigated Definitionen der untersuchten kephalometrischen Variablen

Variable	Meaning	Definition
SNA	SNA (°)	Anteroposterior maxillary position to anterior cranial plane
SNB	SNB (°)	Anteroposterior mandibular position to anterior cranial plane
ANB	ANB (°)	Anteroposterior relation of maxilla and mandible
Ind_ANB	Ind. ANB (°)	Individualized ANB angle according to the formula: Ind. ANB (°)= 35.16 + 0.4(SNA) + 0.2(SN-MeGo)
WITSmmC	Wits (mm)	Length of distance of the intersection points A’ and B’, which are determined by perpendicular lines through A and B to the occlusal plane
NL_NSL	NL-NSL (°)	Inclination of palatal plane in relation to anterior cranial base
ML_NSL	ML-NSL (°)	Inclination of mandibular plane in relation to anterior cranial base
ML_NL	ML-NL (°)	Divergence of mandibular plane and palatal planes
ArGoMe	ArGoMe (°)	Gonial angle
OK1_NL	OK1-NL-Winkel (°)	Axis of maxillary incisor to palatal plane
OK1_NA	OK1-NA (°)	Axis of maxillary incisor in relation to the NA line
UK1_ML	UK1-ML (°)	Axis of mandibular incisor to mandibular plane
UK1_NB	UK1-NB (°)	Axis of mandibular incisor in relation to the NB line
AA_OlpC	AA_Herbst (mm)	Maxillary length
Pog_OLpmmC	Pog_OLp (mm)	Mandibular length
IsOK_OLpmmC	IsOK_Olp (mm)	Anteroposterior position of the maxillary central incisor to the occlusal plane
IsUK_OLpmmC	IsUK_Olp (mm)	Anteroposterior position of the mandibular central incisor to the occlusal plane
Mp6OK_OLpmmC	Mp6OK_OLp (mm)	Anteroposterior position of the maxillary first molar to the occlusal plane
Mp6UK_OLpmmC	Mp6UK_OLp (mm)	Anteroposterior position of the mandibular first molar to the occlusal plane

### Statistical analysis

A descriptive analysis of the different investigated variables was performed. To analyze significant differences over the observed period within each group (T0–T1), a Wilcoxon signed-rank test was carried out. Significant differences between groups were determined by a Mann–Whitney U test. To analyze the treatment effect of the treated groups (TSUS/SUS), the mean values of the variables of the respective control groups were subtracted from the treatment groups. All analyses were performed using SPSS (version 26, IBM, Armonk, NY, USA).

## Results

The patient groups consisted of 22.9% women (77.1% men) in the SUS group and 45.7% women (54.3% men) in the TSUS group. The mean age was 13.7 ± 1.0 years in the SUS group and 13.9 ± 1.0 years in the TSUS group. The observation periods between T0 and T1 were 2.19 years (SUS group), 2.2 years (SUS control group), 2.08 years (TSUS group) and 2.08 years (TSUS control group), respectively.

### Comparison of the absolute values at T0 and T1

**Fig. 2:**
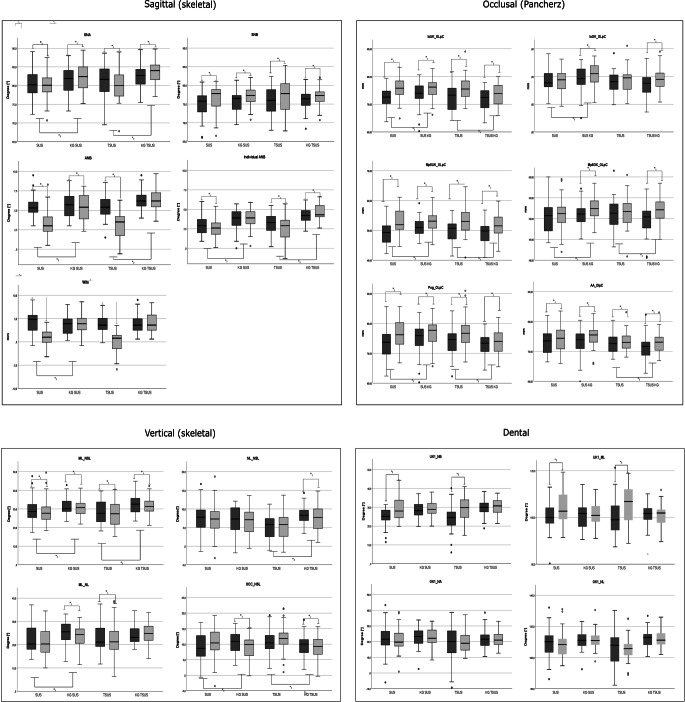
Comparison of initial (T0) and final (T1) cephalometric values of patients treated with a fixed functional appliance without (SUS) or with an additional spring (TSUS) and the respective control groups (KG). A *p*-value of < 0.05 was considered significant. For abbreviations, see Table 2 Vergleich der kephalometrischen Anfangs- (T0) und Endwerte (T1) von Patienten, die mit einer festsitzenden Klasse-II-Mechanik ohne (SUS) oder mit einer zusätzlichen Feder (TSUS) behandelt wurden und der jeweiligen Kontrollgruppen (KG). Ein *p*-Wert von < 0,05 wurde als signifikant angenommen. Abkürzungen s. Tab.2

Figure 2 summarizes the results at T0 and T1 for both the treatment and control groups. Analysis of the sagittal values showed that treatment with the SUS and TSUS resulted in a significant reduction of SNA, ANB, Indiv. ANB angle, and Wits value as well as a significant increase of the SNB angle when comparing T0 and T1. Comparing the ANB to the Indiv. ANB, all groups exhibited a skeletal class II at the beginning of treatment (T0). After treatment (T1), the SUS and TSUS groups achieved a skeletal class I. Significant differences between the SUS/TSUS and the respective control groups could be observed for the parameters SNA, ANB, Indiv. ANB (and for the WITS value in the SUS group). Both the SUS and TSUS group showed significant differences between T0 and T1 related to the position of the mandibular incisors by means of an increase of the parameters UK1-ML and UK1-NB. The maxillary incisors did not show any significant differences from T0 to T1. The analysis according to Pancherz [29] exhibited a significant mesialization of the lower molars in the SUS and in the TSUS group between T0 and T1. In addition, all groups exhibited an increase in mandibular length. In both treatment groups, the maxillary length remained almost unchanged, whereas the control groups showed an increase in maxillary length.

### Analysis of the changes per year

**Fig. 3:**
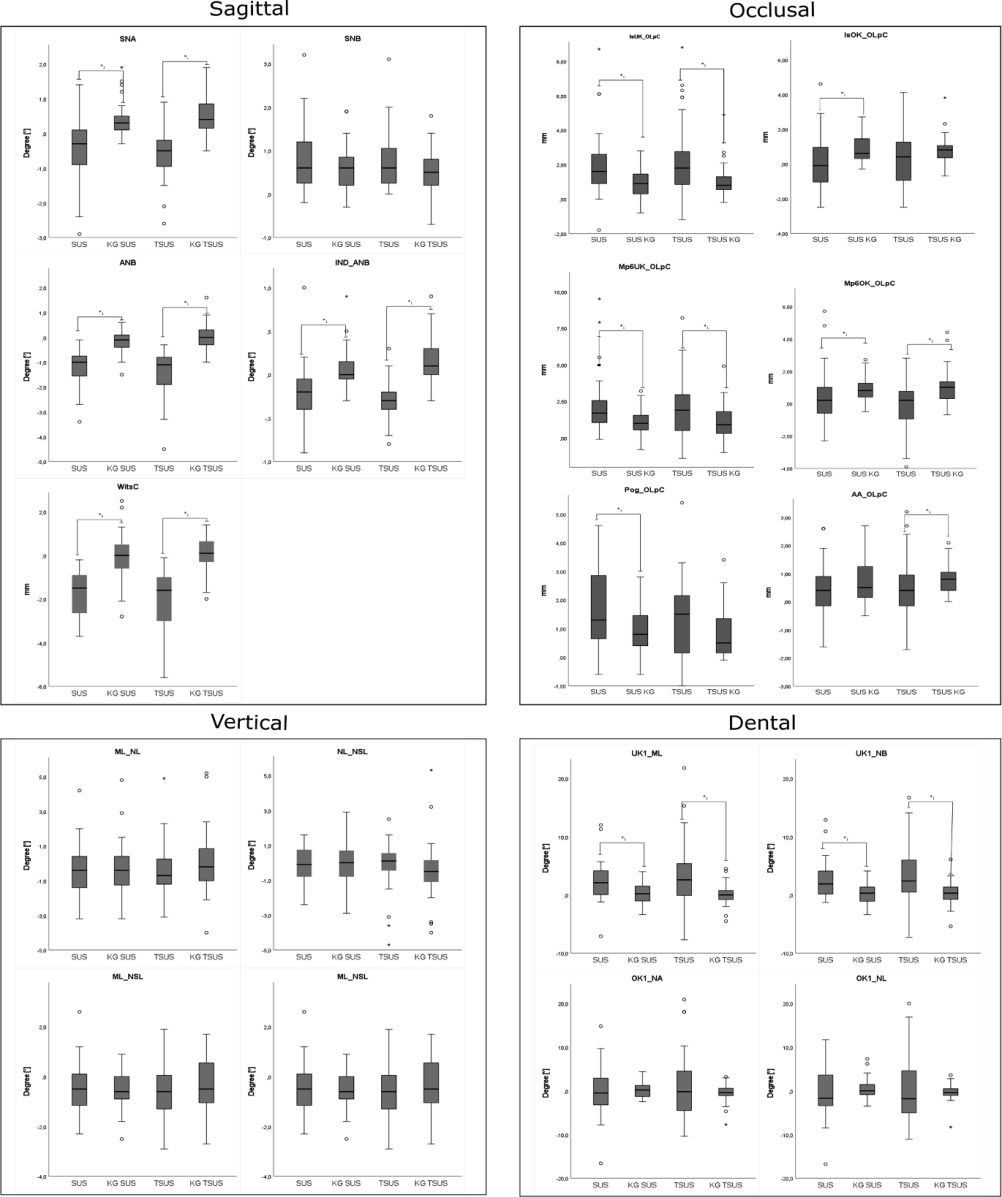
Comparison of mean differences between initial (T0) and final (T1) cephalometric values of patients, treated with a fixed functional appliance without (SUS) or with an additional spring (TSUS) and the respective control groups (KG) per year. A *p*-value of < 0.05 was considered significant. For abbreviations, see Table 2 Vergleich der mittleren Differenzen zwischen kephalometrischen Anfangs- (T0) und Endwerte (T1) von Patienten, die mit einer festsitzenden Klasse-II-Mechanik ohne (SUS) oder mit einer zusätzlichen Feder (TSUS) behandelt wurden und der jeweiligen Kontrollgruppen (KG) pro Jahr. Ein *p*-Wert von < 0,05 wurde als signifikant angenommen. Abkürzungen s. Tab.2

Active treatment duration with the FFA appliances averaged 5.31 months (SUS group) and 4.37 months (TSUS group). In order to estimate and compare the treatment related changes, all values were calculated on a one-year basis (Fig. 3). Comparing the sagittal parameters of the SUS and TSUS groups to their respective control groups, both groups showed a significant increase of the SNA, ANB, Indiv. ANB, and WITS value. The parameter SNB exhibited no significant differences in either of the two treatment groups (SUS/TSUS). No significant differences were also observed for the vertical values between SUS, TSUS, and the respective control groups. Comparing the dental parameters of the SUS and the TSUS group with their respective control groups, a significant increase of the parameters UK1-ML and UK1-NB was shown in both groups. No significant differences were observed for the upper jaw.

According to the analysis of Pancherz [29] treatment with the SUS and TSUS appliances resulted in an increased proclination and an anterior position of the mandibular incisors in contrast to their control group. In addition, a significantly greater increase in mandibular length and a significantly smaller increase in maxillary length were observed. In the upper jaw, the SUS/TSUS groups showed a significantly reduced mesialization of the molars in contrast to their control groups. In the lower jaw, on the other hand, an increased mesialization was observed for the SUS and the TSUS group.

### Treatment effects

**Fig. 4:**
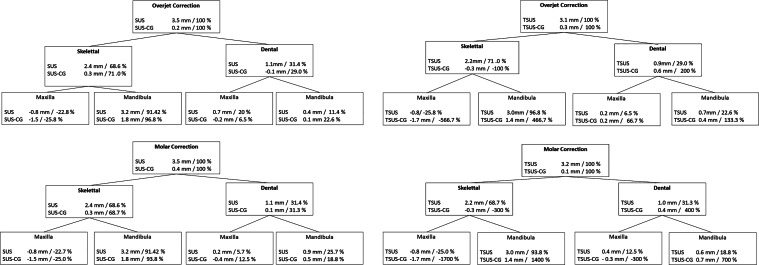
Mechanism of overjet and molar correction treated with a fixed functional appliance (FFA) without (SUS) and with an additional spring (TSUS) and their respective control groups (SUS-CG/TSUS-CG) between T0 and T1. *CG* control group Mechanismus von Overjet- und Molaren-Korrektur bei FFA(„fixed functional appliance“)-Behandlung ohne (SUS) und mit zusätzlicher Feder (TSUS) und ihren jeweiligen Kontrollgruppen (SUS-CG/TSUS-CG) zwischen T0 und T1. *CG* Kontrollgruppe

The mean values for the examined changes between T0 and T1 for the SUS and TSUS groups together with their respective control groups are shown in Table 3. In addition, to estimate the treatment effects, the dental and skeletal changes were related to those of the corresponding control groups (Fig. 4). A greater reduction of the parameters SNA, ANB, WITS, and Indiv. ANB was observed in the TSUS group in contrast to the SUS group. Furthermore, the TSUS group exhibited an increased proclination and anteposition of the mandibular anterior teeth. The Pancherz analysis showed a comparable therapy effect for the SUS and TSUS groups except for the changes of the maxillary anterior teeth.

**Table 3:** Treatment effects in the fixed functional appliance (FFA) treatment without (SUS) and with an additional spring (TSUS). From the mean value of the examined variables between T0 and T1 of the SUS/TSUS group (x), the mean value of the examined variables between T0 and T1 of the respective control groups (x’) was subtracted to estimate the respective treatment effects Therapeutischer Effekt der FFA(„fixed functional appliance“)-Behandlung ohne (SUS) und mit zusätzlicher Feder (TSUS). Vom Mittelwert der untersuchten Variablen zwischen T0 und T1 der SUS/TSUS-Gruppe (x) wurde der Mittelwert der untersuchten Variablen zwischen T0 und T1 der jeweiligen Kontrollgruppen (x’) subtrahiert, um die jeweiligen Therapieeffekte zu ermitteln

		SUS (x)		SUS control (x’)		x–x’	*p*-value^b^	TSUS (x)		TSUS control (x’)		x–x’	*p*-value^b^
		Mean	SD	Mean	SD			Mean	SD	Mean	SD		
*Sagittal*^a^													
SNA (°)	35.0	−0.7	1.5	0.8	1.0	−1.4	< 0.001	−1.1	−1.0	1.0	1.1	−2.1	< 0.001
SNB (°)	35.0	1.5	1.1	1.2	1.0	0.3	0.2	1.3	1.3	1.1	1.0	0.3	0.4
ANB (°)	35.0	−2.1	1.0	−0.4	0.9	−1.7	< 0.001	−2.4	1.1	0.0	0.9	−2.3	< 0.001
Wits (mm)	35.0	−3.3	1.9	0.1	1.9	−3.4	< 0.001	−3.8	2.2	0.3	1.4	−4.1	< 0.001
Ind. ANB (°)	35.0	−0.4	0.6	0.1	0.4	−0.5	< 0.001	−0.6	0.5	0.3	0.4	−0.9	< 0.001
*Vertical*^a^													
ML-NL (°)	35.0	−0.7	2.8	−1.2	2.6	0.6	0.5	−1.2	2.3	0.0	2.6	−1.3	0.1
NL-NSL (°)	35.0	−0.3	2.3	−0.1	2.2	−0.2	1.0	−0.1	1.8	−0.8	2.1	0.8	0.1
ML-NSL (°)	35.0	−0.9	1.6	−1.2	1.7	0.3	0.8	−1.3	2.0	−0.7	1.9	−0.6	0.2
Occ-NSL (°)	35.0	1.1	3.2	−1.4	3.2	2.4	0.01	1.1	3.0	−0.8	2.3	1.9	0.0
*Dental*^a^													
OK1-NSL (°)	35.0	−0.8	10.3	0.8	3.8	−1.6	0.1	0.3	11.8	0.7	3.9	−0.4	0.4
UK1-ML (°)	35.0	4.3	5.7	0.4	3.5	3.9	0.01	5.4	7.3	−0.1	2.7	5.5	0.01
OK1-NA (mm)	35.0	2.4	2.2	0.4	1.4	2.1	1.0	0.7	0.7	−0.2	1.6	0.9	0.3
OK1-NA (°)	35.0	−0.2	10.4	0.2	3.8	−0.4	0.5	1.1	11.8	−0.2	4.0	1.3	0.7
UK1-NB (mm)	35.0	0.9	1.9	0.2	1.0	0.8	0.1	0.4	2.2	0.1	1.1	0.3	0.3
UK1-NB (°)	35.0	4.9	5.1	0.2	3.7	4.7	0.01	5.6	6.9	0.2	2.9	5.4	< 0.001
OK1-NL-Winkel (°)	35.0	−1.1	10.5	0.7	4.2	−1.8	0.2	0.3	11.6	−0.2	3.6	0.5	0.4
*Occlusal (Pancherz)*^a^													
IsOK_Olp (mm)	35.0	−0.1	2.7	1.7	1.6	−1.8	0.3	0.6	1.6	1.6	1.4	−1	0.5
IsUK_Olp (mm)	35.0	3.4	2.4	1.9	2.0	*+1.5*	0.07	3.7	1.9	1.9	1.3	1.8	0.5
Pog_OLp (mm)	35.0	3.2	2.6	1.8	1.9	1.4	0.1	3.0	1.4	1.4	1.3	1.6	0.1
Mp6OK_OLp (mm)	35.0	0.6	2.9	1.9	1.9	−1.3	0.5	0.4	2.0	2.0	1.7	−1.6	0.1
Mp6UK_OLp (mm)	35.0	4.1	2.9	2.3	2.1	1.8	0.6	3.6	2.1	2.1	1.9	1.4	0.4
AA_Herbst (mm)	35.0	0.8	2.1	1.5	1.6	−0.7	0.3	0.9	1.7	1.7	1.2	−0.9	0.06

^a^For abbreviations and definitions of the variables, see Table 2

^b^A *p*-value of < 0.05 was considered as significant

## Discussion

The aim of this retrospective cephalometric study was to analyze the influence of additional force-generating springs on dental and skeletal effects in patients treated with a hybrid FFA.

No statistically significant differences could be observed between the SUS and TSUS groups for any of the cephalometric parameters investigated. Thus, the null hypothesis was rejected, although the estimated treatment effect (Table 3) implied a trend toward increased skeletal and dental effects in the TSUS group.

Post hoc power analysis indicated that a sample size of 200 patients per group would have been required to possibly end up with statistically significant results for the small differences between the SUS and the TSUS group for the variable ANB. Therefore, the differences between the two treatment groups were smaller than the estimated effect based on the studies used to determine sample size [8, 9, 18, 39].

Furthermore, the force differences between the investigated appliances might have been overestimated. Hybrid FFAs use intermaxillary force springs in addition to a telescoping mechanism and spacer rings for progressive mandibular advancement and iterative activity instead of single-step advancement (bite-jumping) [3]. Forces produced by hybrid FFAs force springs usually vary from 1.5 to 2.6 N [24]. In cases with a delayed response to treatment or in cases with little or no residual growth, the use of a larger force has been proposed [7]. In this study, the forces generated by the used springs were 2.5 N (SUS) and 5.5 N (TSUS), respectively. However, this calculation does not take into account the forces acting between the upper and lower arch resulting from the rigid telescoping mechanism. These forces depend on the distance between the maxillary and mandibular attachment, the length of the FFA used, and the application of spacer rings. Although the applied force of the spring in the TSUS group was more than twice than in the SUS group, the resulting effective difference in total force may actually be significantly less.

The mean active treatment duration with the FFAs was short, 5.31 months for the SUS group and 4.37 months for the TSUS group, but other studies on hybrid FFA reported comparable durations (4.53 months [2], 5–7 months [20] and 5 months [25]).

Nevertheless, both treatment groups (SUS and TSUS) exhibited an effective correction of the class II malocclusion, illustrated by a significant reduction of the parameters SNA, and ANB and an increase of the SNB. Thus, a skeletal class I relationship could be achieved in both treatment groups in contrast to the control groups. These findings are in line with the existing literature [10, 18, 20, 25, 40, 41]. Vertical skeletal changes caused by the FFA therapy could not be observed, indicating a good vertical control of the mandibular plane and the occlusal plane, which are also in line with the literature [2, 34, 39, 41]. Dental parameter changes included mainly a protrusion of the lower incisors with a mean increase in UK1-ML of 4.34° (SUS) and 5.38° (TSUS), which is in the range of previous studies [2, 11, 20, 25].

The Pancherz analysis also showed a similar extent and distribution of skeletal and dental effects for the SUS and TSUS groups. The values for molar and overbite corrections were lower than those in some previous studies in which cephalometric radiographs were obtained immediately after appliance removal. However, they are consistent with studies in which immediate posttreatment relapse was accounted for [19, 35], since in this study the cephalometric radiographs were obtained at a later time, before debonding.

### Limitations

Two-dimensional cephalometric analysis is subject to projection errors, distortions, overlay of relevant structures and inaccuracies in reference point identification [23]. However, it has been shown that the results of two-dimensional cephalometric studies are in good agreement with recent 3D morphometric studies regarding class II therapy with FFAs [13]. Due to the retrospective design of this study, the treatment groups (SUS/TSUS) could not be randomized. In addition, the control groups were not from the same patient population as the treatment groups. Finally, despite all efforts to establish a homogeneous study population and suitable control groups, physiological and anatomical differences between the examined groups cannot be excluded. Although gender, growth pattern, and skeletal characteristics were considered for the treatment and control groups, other factors not taken into account could have had an influence on the results.

## Conclusions

No significant statistical differences regarding the cephalometric parameters investigated could be observed between class II treatment with a fixed functional appliance with standard activation (SUS) and with a fixed functional appliance with an additional spring (TSUS). Thus, the null hypothesis was rejected. The application of a higher force magnitude using an additional force-generating spring did not change the amount and distribution of dental and skeletal treatment effects. Both variants were equally effective in treating class II division 1 malocclusions.

## References

[1] Alhammadi MS, Halboub E, Fayed MS, Labib A, El-Saaidi C (2018) Global distribution of malocclusion traits: a systematic review. Dental Press J Orthod 23:40.e41–40.e10. 10.1590/2177-6709.23.6.40.e1-10.onl 10.1590/2177-6709.23.6.40.e1-10.onl PMC6340198 30672991

[2] Aras I, Pasaoglu A (2017) Class II subdivision treatment with the forsus fatigue resistant device vs intermaxillary elastics. Angle Orthod 87:371–376. 10.2319/070216-518.1 27762602 10.2319/070216-518.1 PMC8381987

[3] Aras I, Pasaoglu A, Olmez S, Unal I, Tuncer AV, Aras A (2017) Comparison of stepwise vs single-step advancement with the functional mandibular advancer in class II division 1 treatment. Angle Orthod 87:82–87. 10.2319/032416-241.1 27366817 10.2319/032416-241.1 PMC8388596

[4] Baccetti T, Franchi L, McNamara JA Jr. (2002) An improved version of the cervical vertebral maturation (CVM) method for the assessment of mandibular growth. Angle Orthod 72:316–323. 10.1043/0003-3219 12169031 10.1043/0003-3219(2002)072<0316:AIVOTC>2.0.CO;2

[5] Batista KB, Thiruvenkatachari B, Harrison JE, O’Brien KD (2018) Orthodontic treatment for prominent upper front teeth (class II malocclusion) in children and adolescents. Cochrane Database Syst Rev 3:Cd3452. 10.1002/14651858.CD003452.pub4 29534303 10.1002/14651858.CD003452.pub4 PMC6494411

[6] Bauss O, Röhling J, Schwestka-Polly R (2004) Prevalence of traumatic injuries to the permanent incisors in candidates for orthodontic treatment. Dent Traumatol 20:61–66. 10.1111/j.1600-4469.2004.00230.x 15025687 10.1111/j.1600-4469.2004.00230.x

[7] Baxmann M (2012) Festsitzende Apparaturen zur Klasse-II-Therapie: Bewährte Methoden und neueste Entwicklungen

[8] Baysal A, Uysal T (2013) Soft tissue effects of twin block and Herbst appliances in patients with class II division 1 mandibular retrognathy. Eur J Orthod 35:71–81. 10.1093/ejo/cjq187 21357655 10.1093/ejo/cjq187

[9] Baysal A, Uysal T (2014) Dentoskeletal effects of twin block and Herbst appliances in patients with class II division 1 mandibular retrognathy. Eur J Orthod 36:164–172. 10.1093/ejo/cjt013 24663007 10.1093/ejo/cjt013

[10] Bock NC, von Bremen J, Ruf S (2016) Stability of class II fixed functional appliance therapy—a systematic review and meta-analysis. Eur J Orthod 38:129–139. 10.1093/ejo/cjv009 25820407 10.1093/ejo/cjv009 PMC4914754

[11] Bucci R, Rongo R, Levate C, Michelotti A, Barone S, Razionale AV, D’Anto V (2019) Thickness of orthodontic clear aligners after thermoforming and after 10 days of intraoral exposure: a prospective clinical study. Prog Orthod 20:36. 10.1186/s40510-019-0289-6 31495908 10.1186/s40510-019-0289-6 PMC6732265

[12] Cozza P, Baccetti T, Franchi L, De Toffol L, McNamara JA Jr. (2006) Mandibular changes produced by functional appliances in class II malocclusion: a systematic review. Am J Orthod Dentofacial Orthop 129:599.e1–12. 10.1016/j.ajodo.2005.11.010 (discussion e591–596) 16679196 10.1016/j.ajodo.2005.11.010

[13] Fan Y, Schneider P, Matthews H, Roberts WE, Xu T, Wei R, Claes P, Clement J, Kilpatrick N, Penington A (2020) 3D assessment of mandibular skeletal effects produced by the Herbst appliance. BMC Oral Health 20:117. 10.1186/s12903-020-01108-4 32299402 10.1186/s12903-020-01108-4 PMC7164294

[14] Faul F, Erdfelder E, Lang A‑G, Buchner A (2007) G*Power 3: a flexible statistical power analysis program for the social, behavioral, and biomedical sciences. Behav Res 39:175–191 10.3758/bf03193146 17695343

[15] Flores-Mir C, Ayeh A, Goswani A, Charkhandeh S (2007) Skeletal and dental changes in class II division 1 malocclusions treated with splint-type Herbst appliances. A systematic review. Angle Orthod 77:376–381. https://doi.org/10.2319/0003-3219(2007)077[0376:SADCIC]2.0.CO;2 17319777 10.2319/0003-3219(2007)077[0376:SADCIC]2.0.CO;2

[16] Foncatti CF, Castanha Henriques JF, Janson G, Caldas W, Garib DG (2017) Long-term stability of class II treatment with the Jasper jumper appliance. Am J Orthod Dentofacial Orthop 152:663–671. 10.1016/j.ajodo.2017.03.029 29103444 10.1016/j.ajodo.2017.03.029

[17] Frujeri Mde L, Frujeri JA, Bezerra AC, Cortes MI, Costa ED Jr. (2014) Socio-economic indicators and predisposing factors associated with traumatic dental injuries in schoolchildren at Brasília, Brazil: a cross-sectional, population-based study. BMC Oral Health 14:91. 10.1186/1472-6831-14-91 25037704 10.1186/1472-6831-14-91 PMC4223362

[18] Hanandeh BA, El-Bialy AA (2010) Evaluating the effect of Sabbagh Universal Spring during treatment of growing class II malocclusions. Int J Orthod Milwaukee 21:13–24 21314084

[19] Hansen K (2003) Treatment and posttreatment effects of the Herbst appliance on the dental arches and arch relationships. Semin Orthod 9:67–73. 10.1053/sodo.2003.34026

[20] Hemmatpour S, Mokhtar A, Rakhshan V (2017) Effects of Sabbagh universal spring 2 fixed functional appliance on class II/1 patients at their postpubertal-peak growth period compared with the extraction method : a randomized clinical trial. J Orofac Orthop 78:41–51. 10.1007/s00056-016-0060-2 27858112 10.1007/s00056-016-0060-2

[21] Karbach M, Zöller C, Zöller G, Wehrbein H, Erbe C (2021) The Herbst appliance and its modifications—prevalence and individuality. Head Face Med 17:15. 10.1186/s13005-021-00266-2 33952290 10.1186/s13005-021-00266-2 PMC8097934

[22] Keim RG, Vogels Iii DS, Vogels PB (2020) 2020 JCO study of orthodontic diagnosis and treatment procedures part 1: results and trends. J Clin Orthod 54:581–610 33232286

[23] LeCornu M, Cevidanes LH, Zhu H, Wu CD, Larson B, Nguyen T (2013) Three-dimensional treatment outcomes in class II patients treated with the Herbst appliance: a pilot study. Am J Orthod Dentofacial Orthop 144:818–830. 10.1016/j.ajodo.2013.07.014 24286905 10.1016/j.ajodo.2013.07.014 PMC3999969

[24] Moro A, Borges SW, Spada PP, Morais ND, Correr GM, Chaves CM Jr., Cevidanes LHS (2018) Twenty-year clinical experience with fixed functional appliances. Dental Press J Orthod 23:87–109. 10.1590/2177-6709.23.2.087-109.sar 29898162 10.1590/2177-6709.23.2.087-109.sar PMC6018450

[25] Oztoprak MO, Nalbantgil D, Uyanlar A, Arun T (2012) A cephalometric comparative study of class II correction with Sabbagh universal spring (SUS(2)) and Forsus FRD appliances. Eur J Dent 6:302–310 22904659 PMC3420838

[26] Pacha MM, Fleming PS, Johal A (2016) A comparison of the efficacy of fixed versus removable functional appliances in children with class II malocclusion: a systematic review. Eur J Orthod 38:621–630. 10.1093/ejo/cjv086 26628629 10.1093/ejo/cjv086

[27] Panagiotidis G, Witt E (1977) Der individualisierte ANB-Winkel. J Orofac Orthop 38:408–416. 10.1007/BF02163219

[28] Pancherz H (1979) Treatment of class II malocclusions by jumping the bite with the Herbst appliance. A cephalometric investigation. Am J Orthod 76:423–442. 10.1016/0002-9416(79)90227-6 291343 10.1016/0002-9416(79)90227-6

[29] Pancherz H (1982) Vertical dentofacial changes during Herbst appliance treatment. A cephalometric investigation. Swed Dent J Suppl 15:189–196 6963773

[30] Petti S (2015) Over two hundred million injuries to anterior teeth attributable to large overjet: a meta-analysis. Dent Traumatol 31:1–8. 10.1111/edt.12126 25263806 10.1111/edt.12126

[31] Pfeiffer JP, Grobety D (1975) The class II malocclusion: differential diagnosis and clinical application of activators, extraoral traction, and fixed appliances. Am J Orthod 68:499–544. 10.1016/0002-9416(75)90084-6 1059330 10.1016/0002-9416(75)90084-6

[32] Proffit WR, Fields HW Jr., Moray LJ (1998) Prevalence of malocclusion and orthodontic treatment need in the United States: estimates from the NHANES III survey. Int J Adult Orthodon Orthognath Surg 13:97–106 9743642

[33] Ritto AK, Ferreira AP (2000) Fixed functional appliances—a classification. Funct Orthod 17:12–30 11307418

[34] Ruf S, Pancherz H (1996) The effect of Herbst appliance treatment on the mandibular plane angle: a cephalometric roentgenographic study. Am J Orthod Dentofacial Orthop 110:225–229. 10.1016/s0889-5406(96)70113-0 8760851 10.1016/s0889-5406(96)70113-0

[35] Pancherz H (1997) The effects, limitations, and long-term dentofacial adaptations to treatment with the Herbst appliance. Semin Orthod 3:232–243. 10.1016/s1073-8746(97)80056-4 9573885 10.1016/s1073-8746(97)80056-4

[36] Seehra J, Fleming PS, Newton T, DiBiase AT (2011) Bullying in orthodontic patients and its relationship to malocclusion,self-esteem and oral health-related quality of life. J Orthod 38:247–256. 10.1179/14653121141641 22156180 10.1179/14653121141641

[37] Shaw WC, Addy M, Ray C (1980) Dental and social effects of malocclusion and effectivenessof orthodontic treatment: a review. Community Dent Oral Epidemiol 8:36–45. 10.1111/j.1600-0528.1980.tb01252.x 6989548 10.1111/j.1600-0528.1980.tb01252.x

[38] Silva LF, Thomaz EB, Freitas HV, Pereira AL, Ribeiro CC, Alves CM (2016) Impact of malocclusion on the quality of life of Brazilian adolescents: a population-based study. PLoS ONE 11:e162715. 10.1371/journal.pone.0162715 27690356 10.1371/journal.pone.0162715 PMC5045190

[39] Tomblyn T, Rogers M, Andrews L 2nd, Martin C, Tremont T, Gunel E, Ngan P (2016) Cephalometric study of class II division 1 patients treated with an extended-duration, reinforced, banded Herbst appliance followed by fixed appliances. Am J Orthod Dentofacial Orthop 150:818–830. 10.1016/j.ajodo.2016.04.020 27871709 10.1016/j.ajodo.2016.04.020

[40] Wichelhaus (2017) Orthodontic therapy fundamental treatment concepts. Color Atlas of dental medicine, 1st edn. Thieme

[41] Zymperdikas VF, Koretsi V, Papageorgiou SN, Papadopoulos MA (2016) Treatment effects of fixed functional appliances in patients with class II malocclusion: a systematic review and meta-analysis. Eur J Orthod 38:113–126. 10.1093/ejo/cjv034 25995359 10.1093/ejo/cjv034 PMC4914762

